# Total Mercury Levels in Sea Cucumbers *Holothuria polii* and *Holothuria tubulosa* and Sediments from Two Sites of Southern Italy

**DOI:** 10.1007/s00128-026-04288-x

**Published:** 2026-08-28

**Authors:** Gabriella Di Lena, Irene Casini, Roberto Caproni

**Affiliations:** Consiglio per la ricerca in agricoltura e l’analisi dell’economia agraria (CREA), Centro di ricerca Alimenti e Nutrizione, Via Ardeatina 546, 00178 Roma, Italia

**Keywords:** Echinoderms, Holothurians, Contaminants, Heavy metals, Bioaccumulation, Bioconcentration

## Abstract

Holothurians are increasingly recognized as bioindicators and bioremediators in marine ecosystems. This study assessed the relationship between total mercury (THg) levels in *Holothuria polii* and *Holothuria tubulosa* from two Southern Italian coastal sites differing for ecological and anthropogenic characteristics, and those in corresponding marine sediments. THg levels in holothurians were consistently low across species and sites (0.0022–0.0146 mg kg^− 1^ wet mass) and showed no direct correspondence with sediment levels, which were high (1.5080 ± 1.1687 mg kg^− 1^ dry mass) at the Ionian site (IS), and low (0.0055 ± 0.0028 mg kg^− 1^ dry mass) at the Adriatic site (AS). Despite lower sediment mercury contamination, the bioconcentration factor, indicator of the efficiency of metal bioaccumulation was higher at the AS site (3.54–4.76) than at the IS site (0.021–0.022), suggesting different mercury bioavailability in the sites. Size-related analyses of THg in holothurians indicated that THg levels in individual holothurians correlated positively with body size (*p* < 0.05), except for *H. tubulosa* from AS. Results demonstrate that site-specific factors, more than species-specific ones, play a key function in controlling mercury bioavailability and uptake in the studied holothurians, supporting their potential role in environmental monitoring and their safe consumption in the studied areas.

**Supplementary Information:**

The online version contains supplementary material available at https://doi.org/10.1007/s00128-026-04288-x.

## Introduction

Holothurians are benthic echinoderms widely distributed in marine ecosystems, from shallow coastal zones to the deep sea. Their ecological role as deposit feeders, ingesting sediment and organic detritus, makes them key agents in sediment bioturbation and nutrient cycling (Purcell et al. 2016). In recent years, holothurians have attracted increasing attention for their ecological roles as bioremediators of benthic ecosystems, their application in integrated multitrophic aquaculture systems (Rakaj et al. 2018, 2019), and their potential as bioindicators of environmental contamination, particularly regarding heavy metals (Boluda-Botella et al. 2023; González-Delgado et al. 2024; Bhuyan et al. 2024).

Among the several pollutants affecting marine environments, mercury stands out due to its high toxicity, persistence and ability to bioaccumulate and biomagnify through trophic webs, posing risks to both ecosystem and human health. Mercury contamination in marine systems is often linked to both natural geochemical processes and anthropogenic sources such as industrial discharge, mining, and agricultural runoff (Gworek et al. 2016). In coastal regions, sediments act as primary reservoirs for mercury, where it can remain bound or be remobilized under changing environmental conditions, posing long-term risks to benthic organisms (Koron and Faganeli 2012; Gworek et al. 2016).

In the Mediterranean Sea, holothurian species such as *Holothuria polii* and *Holothuria tubulosa* have been object of studies due to their market interest, ecological relevance and exposure to heavy metals (González-Wangüemert et al. 2018; Sales et al. 2023). Studies examining mercury levels in sea cucumbers report low and variable concentrations compared to predatory fish, further supporting the link between low trophic levels and low mercury accumulation (Noël et al. 2011; Elvevoll et al. 2022). Nevertheless, due to their close association with sediments, where pollutants may accumulate, as well as their low mobility and long lifespan, holothurians have been proposed as effective bioindicators of sediment and environmental contamination, with the potential to play a key role in monitoring heavy metal levels in marine ecosystems (Bhuyan et al. 2024; González-Delgado et al. 2024). This sentinel role is particularly relevant in the Mediterranean, where coastal ecosystems are increasingly impacted by anthropogenic pressure and sediment quality is a critical determinant of ecological integrity (Hosseini et al. 2022; Özbay 2024).

In a previous investigation on the compositional and nutritional profiles of *Holothuria polii* and *Holothuria tubulosa*, we reported consistently low mercury concentrations in both species across two Southern Adriatic and Ionian sites distinguished by different ecological and anthropogenic characteristics (Di Lena et al. 2025). The present study was undertaken to expand and refine previous findings by assessing the relationship between mercury levels in *Holothuria polii* and *Holothuria tubulosa* and those in marine sediments, while also accounting for site-specific ecological and anthropogenic characteristics. The overall aim is to improve the understanding of the potential of these holothurian species as reliable bioindicators of mercury contamination in different environmental contexts. By combining mercury concentrations measured in holothurians and sediments, this study provides additional up-to-date insights into mercury distribution dynamics in these deposit‑feeding Mediterranean species from different coastal settings. In addition, size related patterns of mercury accumulation were examined to assess the consistency of low bioaccumulation across the different size classes and to provide complementary information relevant to consumer safety, given the increasing interest in holothurians as a food resource.

## Materials and Methods

### Study Area

**Fig. 1:**
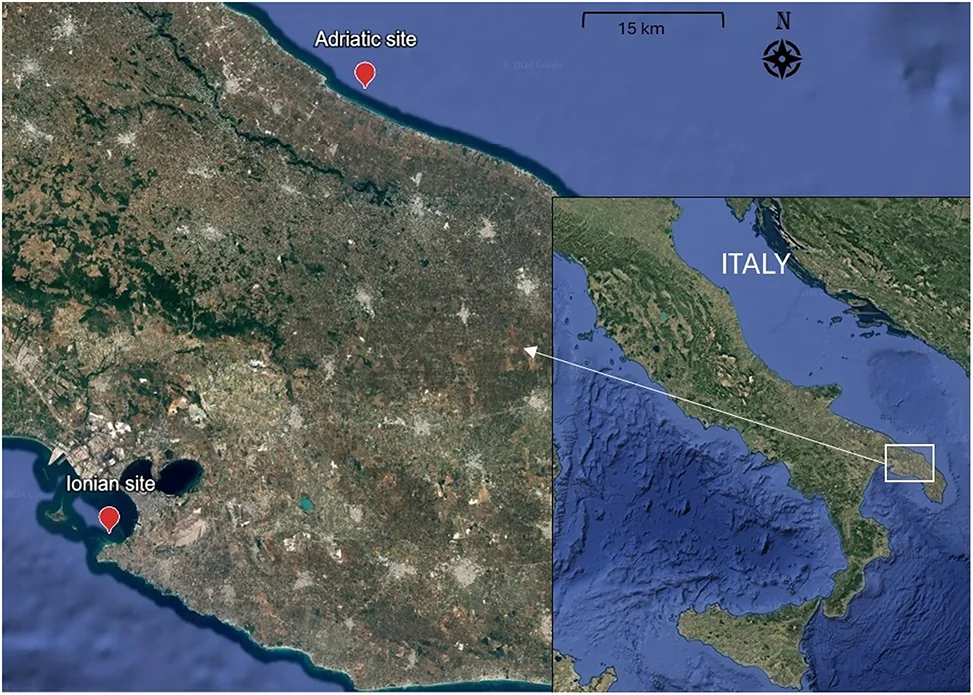
Map of the study area showing the two sampling sites (source: Google Earth)

Sea cucumbers *Holothuria polii* and *Holothuria tubulosa* were collected in two sites of the Apulian coast (Southern Italy), (Fig. 1). One site, located along the Adriatic Sea (AS) near Torre Canne, Brindisi (40°49’39.84"N; 17°31’26.70"E), is characterized by modest anthropogenic pressure and moderate human use. The area is surrounded by predominantly natural and semi natural coastal environments with human activities mainly related to tourism and small-scale fisheries. The second site, located in the Ionian Sea (IS) at Mar Grande, Taranto (40°25′41.52″N; 17°13′24.60″E), represents a highly exploited marine system. This area hosts long-established aquaculture activities, particularly mussel farming, alongside major industrial installations, including a steel manufacturing plant, a petroleum refinery, and a large harbour system, within a densely populated urban setting.

### Sampling

Sea cucumbers and sediments were collected between June and November 2020 by scuba divers. Taxonomic identification of *Holothuria polii* and *Holothuria tubulosa* was conducted through the observation of morphological characteristics and the use of standard dicothomous keys (Rakaj and Fianchini 2024). After collection, sea cucumbers were cleaned, given an identification code, weighed and immediately frozen and transported to CREA Centro di ricerca Alimenti e Nutrizione, Rome, where they were stored at − 80 °C until analyses, carried out within 1 week. A total of 56 specimens belonging to the species *H. polii* (*n* = 28 from the AS, total body weight 109.5 ± 60.4 g and *n* = 28 from the IS, total body weight 65.7 ± 39.4 g) and 41 belonging to *H. tubulosa* (*n* = 20 from the AS, total body weight 203.9 ± 153.5 g and *n* = 21 from the IS, total body weight 112.3 ± 48.2 g) were submitted to instrumental analysis. The body weight of individual specimens from both species and sampling sites is reported in the Supplementary Materials (Table S1).

The day of analyses, sea cucumbers were thawed and the body wall longitudinally excised with a surgical scalpel to remove viscera. Small tissue portions were excised from the central-median axis of the body wall of each specimen and immediately analysed for mercury content. All operations occurred under clean conditions, taking care to avoid any intra- and inter-individual contamination.

Marine surface sediments (top 5 cm) were collected with a stainless-steel grab dredger at each site of sea cucumber collection, frozen (− 20 °C) in polyethylene test tubes and transported to CREA laboratory, where they were freeze-dried (Labogene, Scanvac Coolsafe, Allerød, Denmark) for further analyses.

### Total Mercury (THg) Determination

THg content in sea cucumbers and marine sediments was determined by Thermal Decomposition–Amalgamation–Atomic Absorption Spectrophotometry (DMA-80 Direct Mercury Analyzer, Milestone Srl, Sorisole, BG, Italy). Instrument calibration and analytical procedures were conducted according to the US Environmental Protection Agency Method 7473 as described before (Di Lena et al. 2018).

The limit of detection (LOD) and limit of quantification (LOQ) were determined as 3 and 10 times, respectively, the standard deviation of 10 measurements of reagent blanks divided by the slope of the calibration curve. Values were the following: LOD 0.028 µg kg^− 1^, LOQ 0.093 µg kg^− 1^. Two Certified Standard Reference Materials, CRM 463 Tuna fish muscle (Community Bureau of Reference, European Commission, Luxembourg) and SRM 1570a Spinach Leaves (National Institute of Standards and Technology, Gaithesburg, Maryland, USA) were used for method validation and quality control of data. The values obtained from the analysis of four replicates of reference materials were as follows: CRM 463, certified value 2.85 ± 0.16 mg kg^− 1^, found value 2.67 ± 0.05 mg kg^− 1^, recovery 93.5%; SRM 1570a, certified value 0.030 ± 0.003 mg kg^− 1^, found value 0.029 ± 0.002 mg kg^− 1^, recovery 97.3%.

Marine sediments were analysed without any treatment except lyophilization. Multiple sediment aliquots (> 40) were analysed to ensure representativeness of data. Analyses on sea cucumbers were conducted on single individuals. On average, 5 replicate analyses were accomplished for each sea cucumber.

### Bioconcentration Factor

The Bioconcentration Factor (BCF) was calculated according to the formula: BCF= [THg_tissue_]/[THg_sed_], where THg_tissue_ was the total mercury concentration (mg kg^− 1^ dry mass) in the body wall of sea cucumbers at a given site and THg_sed_ (mg kg^− 1^ dry mass) was the concentration in the corresponding sediment (Aydin-Onen et al. 2015).

### Statistical Analysis

Data are expressed as means ± standard deviations of the THg levels detected in the body wall of sea cucumbers processed and analysed individually, and in marine sediments collected at the corresponding sites. The normality of distributions and homogeneity of variances of THg levels detected in holothurians and sediments were assessed using the Shapiro–Wilk’s and Levene’s tests, respectively. When these assumptions were not met, as in the case of sediment levels, the statistical differences between sites were tested non-parametrically by the Mann-Whitney W test. The non-parametric ANOVA equivalent (Kruskal–Wallis test) was used to compare THg levels in holothurians across the different species and sites, as the normality of distribution was not confirmed. Statistical significance was defined as *p* ≤ 0.05. To determine the correlation between THg concentration and body weight of individual sea cucumbers the Pearson’s correlation coefficient was calculated.

The softwares used for statistical analyses were Statgraphics^®^ Centurion, Version XV (StatPoint Technologies Inc., Warrenton, VA, USA) and Past, version 2.17 (Øyvind Hammer, Natural History Museum, University of Oslo).

## Results and Discussion

**Fig. 2:**
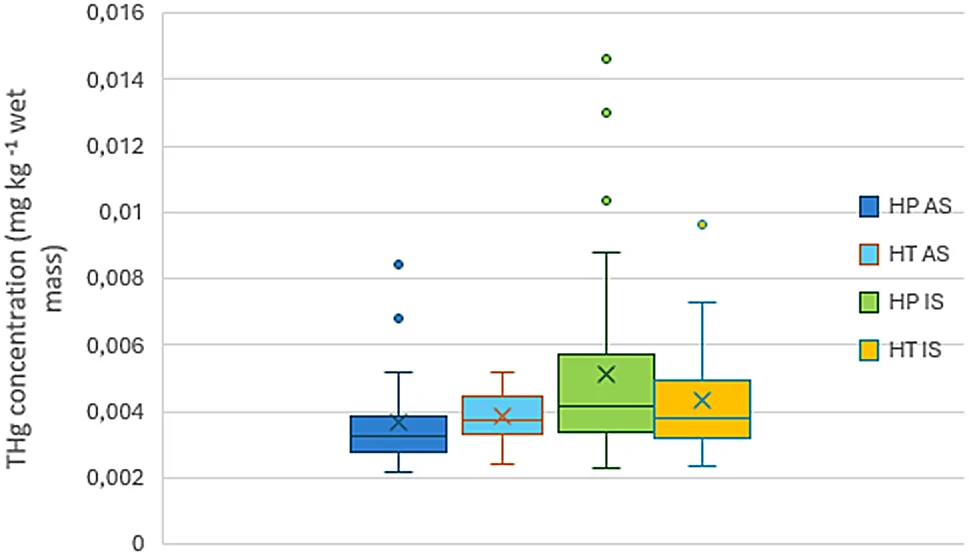
Total Mercury (THg) levels (mg kg wet mass^-1^) in *H. polii* (HP) and *H. tubulosa* (HT) from the Adriatic (AS) and Ionian (IS) sites

The distribution of THg levels across the four holothurian groups is visualized in Fig. 2. *H. polii* levels were in the range 0.0022–0.0084 mg kg^− 1^ and 0.0023–0.0146 mg kg^− 1^ wet mass basis in samples from the AS and IS, respectively. *H. tubulosa* from the two sites showed similar values, with ranges comprised between 0.0024 mg kg^− 1^ and 0.0052 mg kg^− 1^ in samples from the AS site and between 0.0024 mg kg^− 1^ and 0.0096 mg kg^− 1^ wet mass in samples from the IS site. THg concentrations measured in individual specimens from each group are reported in Supplementary Materials (Table S1). Figure 2 highlights differences in central tendency and dispersion among groups. Notably, only *H. tubulosa* from AS exhibited a distribution consistent with normality (*p* > 0.05), while the others showed varying degrees of asymmetry (*p* < 0.05). Statistically significant differences among the groups (*p* < 0.05) were detected. *H. polii* from IS had the highest median and the widest interquartile range of THg, accompanied by some high-value outliers, suggesting a greater variability, potentially reflecting individual responses to localized contamination or episodic exposure to mercury.

This pattern likely reflects environmental characteristics of the IS site, marked by reduced hydrodynamic activity and high anthropogenic pressure, both potentially leading to increased THg accumulation in *H. polii*. In contrast, the more stable and oxygenated conditions of the AS site likely contributed to the narrower distributions observed in both species, with *H. tubulosa* from AS in particular showing a symmetric and compact distribution, possibly due to species-specific resilience combined with favourable environmental conditions. These findings highlight the combined influence of biological traits and site-specific environmental pressures in shaping the observed responses.

The analysis of THg in marine sediments sampled in correspondence of the holothurians collection sites showed contrasting values (Table 1). Sediments from the AS site had low THg concentrations (0.0055 ± 0.0028 mg kg^− 1^ dry mass), while sediments from the IS site exhibited high and variable concentrations (1.5080 ± 1.1687 mg kg^− 1^ dry mass).

**Table 1:** Total mercury levels (mg kg dry mass^-1^) in sediment samples collected at the Adriatic and Ionian sites of holothurian sampling

	Adriatic site			Ionian site			W	*p* value
	Mean ± sd	Min	Max	Mean ± sd	Min	Max		
Sediment	0.0055 ± 0.00283	0.0034	0.0231	1.5080 ± 1.1687	0.5860	7.2659	0.0	0.000*

*Mann-Whitney’s W test: significance of differences between sites at *p* < 0.05

The uniformly low sedimentary THg levels observed at the AS site align with its minimal anthropogenic pressure and active hydrodynamics. A dynamic sediment–water exchange and active water column mixing at this site contribute to homogeneous exposure conditions, consistent with the narrow and symmetric THg distributions observed in both holothurian species from AS site, particularly in *H. tubulosa.* In contrast, the high THg concentrations detected in sediments from the IS site are consistent with the high industrial settlement and low hydrodynamics of the site. These factors are likely to favour mercury accumulation and spatially uneven exposure conditions, consistent with the higher median values, wider dispersion, and presence of outliers observed in *H. polii* from IS (Fig. 2).

Marine sediments, particularly in industrialised areas, are known to reflect mercury concentrations in the water column and act as long-term reservoirs, with mercury mobility and bioavailability influenced by environmental and anthropogenic factors (Birch 2017).

The THg patterns detected in sediments from this study are in line with previous studies highlighting the role of hydrodynamic conditions, sediment characteristics and anthropogenic activities in influencing contaminant levels in sea sediments. As regards the Southern Adriatic Sea area, the low mercury contamination observed in sediments in this study is consistent with previous findings, with sediment grain size and organic matter distribution emerging as key factors controlling mercury concentrations (Droghini et al. 2019; Fanelli et al. 2025).

As regards IS sediments, the high and heterogeneous THg concentrations observed are consistent with previous studies describing the Taranto Gulf as a low‑energy system characterized by scarce hydrodynamism, limited water exchange, and strong anthropogenic inputs where sediments act as long‑term contaminant reservoirs and display marked spatial variability (Cardellicchio et al. 2009). Within the same context, sediments have also been identified as the main sink for mercury, while the transfer to biota is strongly modulated by sediment–water biogeochemical processes, presence of insoluble sulphide forms and speciation in addition to total sedimentary concentrations (Spada et al. 2012; Emili et al. 2016).

The low mercury levels observed in holothurians in this study are consistent with other studies (León et al. 2021; Montero et al. 2021). Overall, the mercury levels observed in holothurians from this study were well below the threshold of 0.5 mg kg^− 1^ for fishery products set by European legislation (European Union Commission 2022), in line with their low trophic level, but apparently not correlated to sediment mercury levels. This lack of correlation is remarkable and unexpected as seemingly contrasts with studies that identify holothurians as sentinel organisms for heavy metal pollution (Bhuyan et al. 2024; González-Delgado et al. 2024). A similar observation, with tissue concentrations of mercury not correlated with those in the sediment, was also reported by Tamburini et al. (2022) in a study on *Holothuria atra* from Indonesia. This discrepancy was linked to differences in the bioavailability of trace elements, influenced by site-specific environmental conditions, water chemistry, sedimentary regimes, and the geochemical speciation of the elements.

The relationship between mercury concentrations in holothurians and in the corresponding sediments may be assessed through the calculation of the bioconcentration factor (BCF), an indicator of the efficiency of metal bioaccumulation in aquatic organisms relative to sediment concentrations. BCF values higher than 1 indicate mercury bioaccumulation, whereas values below 1 indicate no bioaccumulation (Aydin-Onen et al. 2015).

**Fig. 3:**
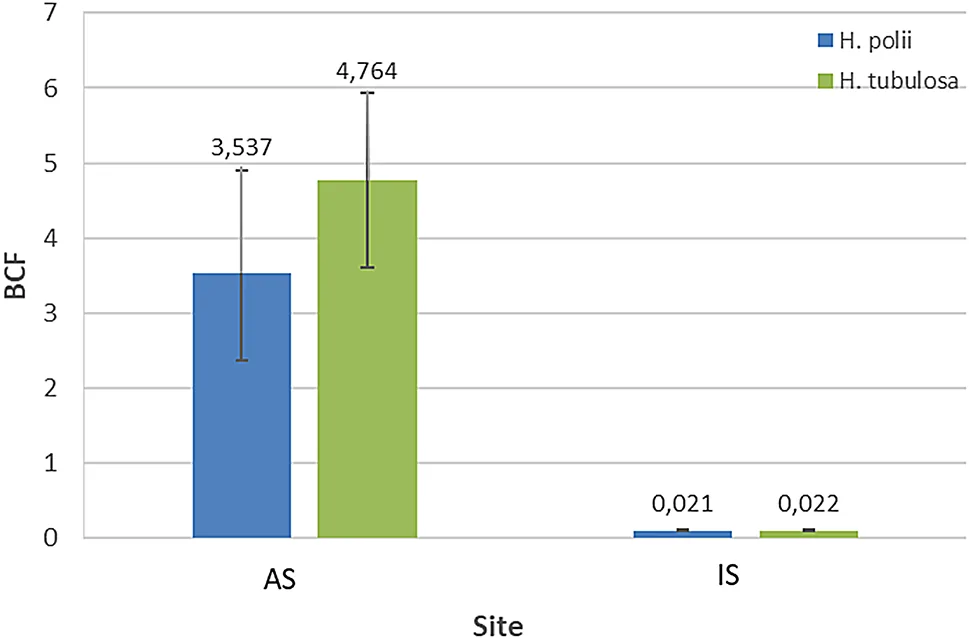
Bioconcentration factor for total mercury in *H. polii* and *H. tubulosa* from the Adriatic (AS) and Ionian sites (IS)

The BCF values calculated for sea cucumbers in this study are reported in Fig. 3. Values were consistently high at the AS site for both species (3.54 ± 1.36 for *H. polii* and 4.76 ± 1.17 for *H. tubulosa*) and low at the IS site (0.021 ± 0.01 for *H. polii* and 0.022 ± 0.01 for *H. tubulosa*). Notably, these values indicate a strong mercury bioaccumulation in sea cucumbers from the AS site, which has the lowest sediment mercury concentration. This may likely reflect the older age of the holothurians, as inferred from the biometric data, but also a higher mercury bioavailability at this site, an aspect deserving further studies. In contrast, almost no bioaccumulation was observed at the IS site, where sediment contamination is higher. These findings highlight the importance of site-specific factors such as sediment properties, anthropogenic pressure, sediment mercury speciation and hydrodynamic conditions, in influencing mercury uptake, rather than species-specific traits. In low-energy environments such as the IS site, mercury may be strongly bound to fine-grained sediments or refractory organic matter, reducing its mobility and biological availability. Under these conditions, mercury can remain largely sequestered within the sediment matrix despite high bulk concentrations (Hsieh and Bugna 2025).

**Fig. 4:**
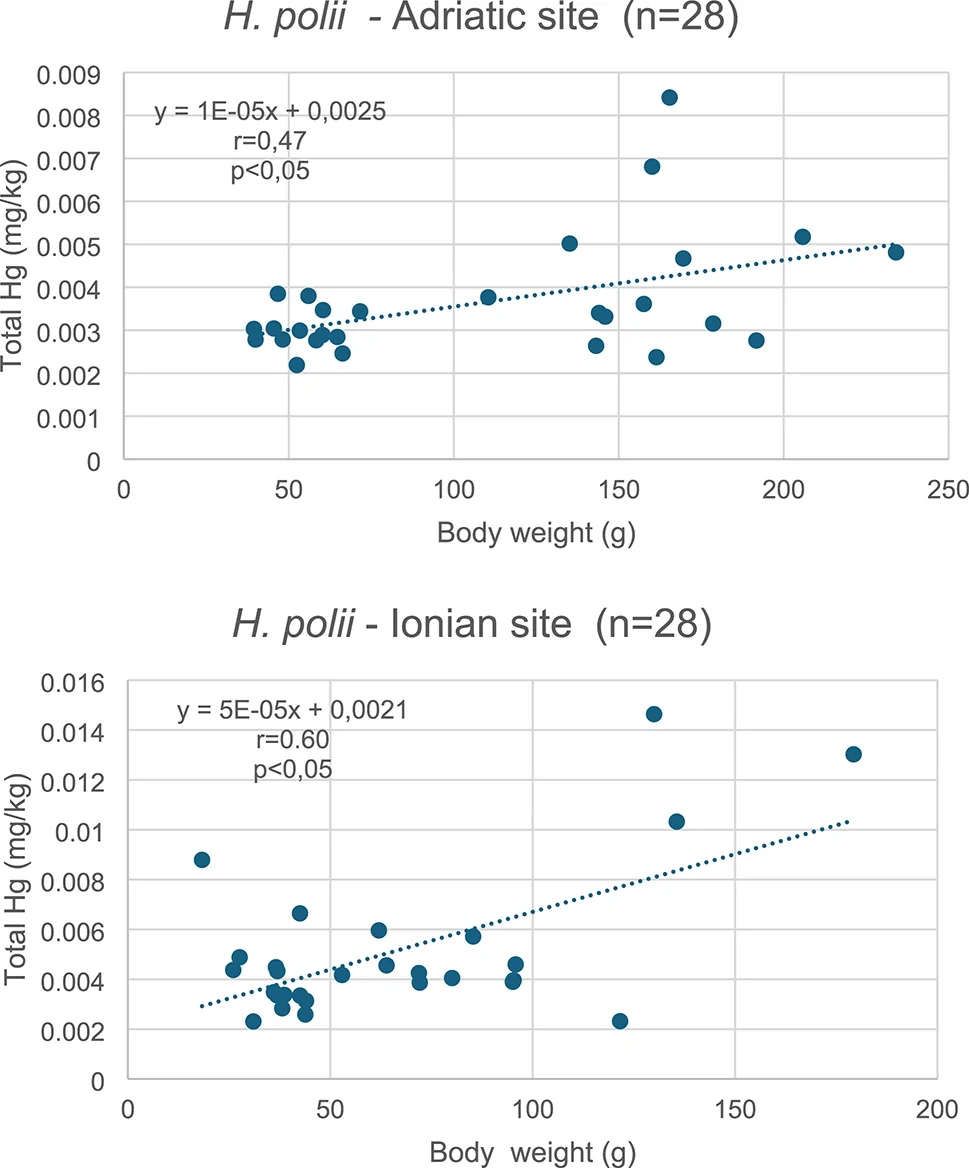
Correlation between total mercury (THg) content (mg kg wet mass^−1^) and body weight (g) of sea cucumbers *H. polii* from the Adriatic and Ionian sites

**Fig. 5:**
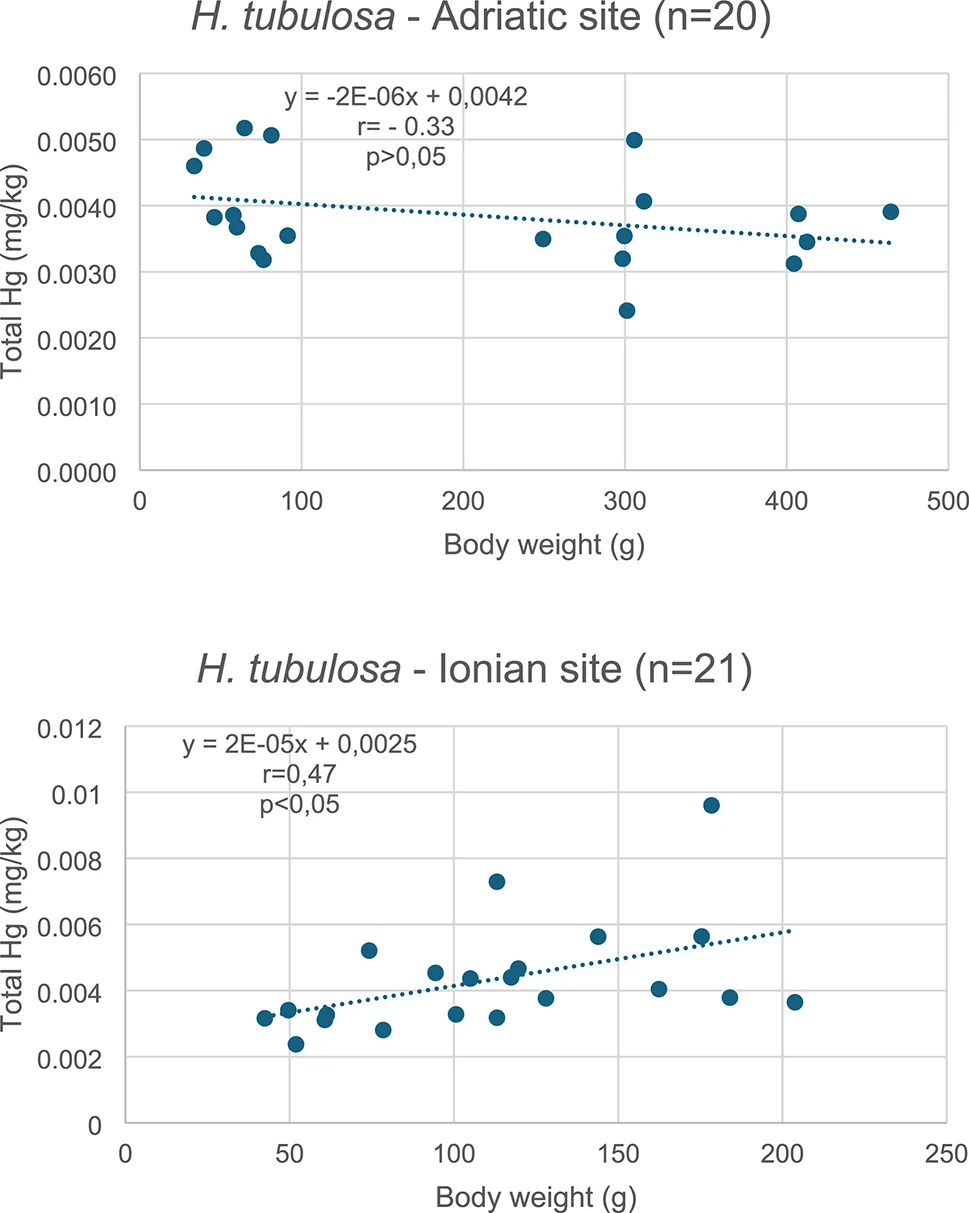
Correlation between total mercury (THg) content (mg kg wet mass^− 1^) and body weight (g) of sea cucumbers *H. tubulosa* from the Adriatic and Ionian sites

To verify the consistency of mercury bioaccumulation across different holothurian sizes, we investigated the relationship between mercury concentration and body size of *Holothuria polii and Holothuria tubulosa*. *H. polii* samples from either site (Fig. 4a, b) showed a positive and significant correlation (*p* < 0.05) between THg content and body size (*r* = 0.47 from AS and *r* = 0.60 from IS), as expected due to the known accumulation of mercury in tissues as a function of time. For *H. tubulosa* (Fig. 5a, b), a positive and statistically significant correlation (*r* = 0.47, *p* < 0.05) was observed between THg content and body size in IS samples. In contrast, in AS samples the correlation was negative but not significant (*r* = − 0.33, *P* > 0.05). This pattern likely reflects the co-occurrence of different factors, such as low site contamination and somatic growth dilution, occurring when efficient growth leads to lower tissue mercury concentration (Chételat et al. 2021). The chemical form and bioavailability of mercury in sediments (inorganic Hg vs. methylmercury), along with dietary sources, strongly influence its accumulation in organisms and determine whether somatic growth dilution can effectively reduce tissue concentrations.

## Conclusions

The findings of this study highlight the importance of site-specific factors such as anthropogenic pressure and hydrodynamic conditions, more than species-specific traits, in influencing mercury uptake and bioavailability for holothurians. The integrated evaluation of mercury concentrations in holothurians and sediments from the two sites, together with their respective bioconcentration factors and size‑related accumulation patterns, provides additional insights into mercury distribution in these deposit‑feeding species. In particular, the present results suggest that sediment characteristics and mercury speciation play a crucial role, deserving further investigation and constant monitoring to better understand the relationship between mercury levels in holothurians and environmental mercury dynamics. Overall, the low mercury concentrations measured in holothurians confirm their limited exposure risk in the studied areas and support their safe consumption. At the same time, these results reinforce the ecological relevance of holothurians as sentinels of site‑specific mercury bioavailability, highlighting their potential role in environmental monitoring and sustainable seafood practices.

## Electronic Supplementary Material

Below is the link to the electronic supplementary material.

Table S1 Body weight (g) and total mercury (THg) contents (mg kg^-1^ wet mass and mg kg^-1^ dry mass) of single holothurians *H. polii* and * H. tubulosa* sampled from the Adriatic (AS) and Ionian sites (IS)
