# Supplementary material for: Total Mercury Levels in Sea Cucumbers Holothuria polii and Holothuria tubulosa and Sediments from Two Sites of Southern Italy
Source: Bull Environ Contam Toxicol. 2026 Aug 28;117(3):50. doi: 10.1007/s00128-026-04288-x (PMC13524999; doi:10.1007/s00128-026-04288-x)
Supplement: Supplementary file 1 [file 128_2026_4288_MOESM1_ESM.pdf]

SUPPLEMENTARY MATERIAL

Total Mercury Levels in Sea Cucumbers *Holothuria polii* and *Holothuria tubulosa* and Sediments from Two Sites of Southern Italy

Gabriella Di Lena*, Irene Casini, Roberto Caproni

Consiglio per la ricerca in agricoltura e l'analisi dell'economia agraria (CREA), Centro di ricerca Alimenti e Nutrizione, Via Ardeatina 546, 00178 Roma, Italia

Table S1 Body weight (g) and total mercury (THg) contents (mg kg^{-1} wet mass and mg kg^{-1} dry mass) of single holothurians *H. polii* and *H. tubulosa* sampled from the Adriatic (AS) and Ionian sites (IS)

	HP-AS			HP-IS			HT-AS			HT-IS		
ID	Body weight	THg (wet mass)	THg (dry mass)	Body weight	THg (wet mass)	THg (dry mass)	Body weight	THg (wet mass)	THg (dry mass)	Body weight	THg (wet mass)	THg (dry mass)
1	169.54	0.0047	0.0247	63.90	0.0046	0.0294	311.68	0.0041	0.0263	94.40	0.0045	0.0343
2	205.75	0.0052	0.0273	52.90	0.0042	0.0270	298.54	0.0032	0.0207	113.10	0.0032	0.0240
3	160.10	0.0068	0.0360	179.30	0.0130	0.0842	305.76	0.0050	0.0322	117.40	0.0044	0.0332
4	143.12	0.0026	0.0139	121.60	0.0023	0.0150	412.55	0.0035	0.0223	143.90	0.0056	0.0425
5	145.89	0.0033	0.0175	130.00	0.0146	0.0946	249.3	0.0035	0.0226	175.40	0.0056	0.0426
6	144.01	0.0034	0.0180	80.10	0.0041	0.0262	404.48	0.0031	0.0186	178.40	0.0096	0.0725
7	234.04	0.0048	0.0254	95.10	0.0039	0.0252	301.21	0.0024	0.0144	184.00	0.0038	0.0286
8	178.61	0.0032	0.0172	71.90	0.0043	0.0254	299.59	0.0035	0.0211	162.40	0.0040	0.0307
9	165.40	0.0084	0.0459	72.10	0.0039	0.0231	407.21	0.0039	0.0231	203.80	0.0036	0.0277
10	110.45	0.0038	0.0205	95.80	0.0046	0.0274	464.37	0.0039	0.0233	119.60	0.0047	0.0354
11	191.71	0.0028	0.0150	95.30	0.0040	0.0236	57.78	0.0039	0.0275	113.10	0.0073	0.0554
12	135.12	0.0050	0.0273	135.60	0.0103	0.0617	46.05	0.0038	0.0273	105.00	0.0044	0.0332
13	161.42	0.0024	0.0129	62.00	0.0060	0.0356	39.66	0.0049	0.0347	128.00	0.0038	0.0286
14	157.58	0.0036	0.0197	85.30	0.0057	0.0341	76.41	0.0032	0.0227	74.20	0.0052	0.0396
15	60.34	0.0035	0.0177	36.90	0.0034	0.0217	91.33	0.0035	0.0253	61.29	0.0033	0.0236
16	66.28	0.0025	0.0126	38.13	0.0028	0.0183	64.7	0.0052	0.0385	100.63	0.0033	0.0237
17	46.68	0.0038	0.0196	31.00	0.0023	0.0149	33.58	0.0046	0.0342	42.44	0.0032	0.0229
18	52.41	0.0022	0.0112	43.83	0.0026	0.0167	81.25	0.0051	0.0376	49.60	0.0034	0.0265
19	48.13	0.0028	0.0142	18.31	0.0088	0.0568	73.3	0.0033	0.0244	78.47	0.0028	0.0219
20	55.94	0.0038	0.0194	38.62	0.0034	0.0218	60.01	0.0037	0.0273	51.92	0.0024	0.0185
21	39.40	0.0030	0.0155	42.51	0.0066	0.0429	-	-		60.71	0.0031	0.0242
22	53.33	0.0030	0.0163	36.92	0.0043	0.0259	-	-		-	-	
23	64.70	0.0028	0.0155	36.57	0.0045	0.0268	-	-		-	-	
24	39.90	0.0028	0.0152	44.00	0.0031	0.0187	-	-		-	-	

25	45.45	0.0030	0.0166	42.54	0.0033	0.0200	-	-		-	-	
26	58.30	0.0028	0.0151	26.02	0.0044	0.0261	-	-		-	-	
27	60.18	0.0029	0.0158	27.60	0.0049	0.0291	-	-		-	-	
28	71.60	0.0034	0.0187	36.08	0.0035	0.0208	-	-		-	-	
**MEAN**	**109.48**	**0.0037**	**0.0195**	**65.71**	**0.0051**	**0.0319**	**203.94**	**0.0039**	**0.0262**	**112.27**	**0.0043**	**0.0328**
SD	*60.41*	*0.0014*	*0.0075*	*39.41*	*0.0030*	*0.0196*	*153.54*	*0.0007*	*0.0064*	*48.23*	*0.0017*	*0.0126*
MIN	39.40	0.0022	0.0112	18.31	0.0023	0.0149	33.58	0.0024	0.0144	42.44	0.0024	0.0185
MAX	234.04	0.0084	0.0459	179.30	0.0146	0.0946	464.37	0.0052	0.0385	203.80	0.0096	0.0725
